# Identification and assessment of stress and associated stressors among veterinary students in India using a cross-sectional questionnaire survey

**DOI:** 10.3389/fpubh.2022.1059610

**Published:** 2022-11-21

**Authors:** Kushal Grakh, Diksha Panwar, Vijay Jayawant Jadhav, Rajesh Khurana, Dheeraj Yadav, Yogesh Chandrakant Bangar, Lokender Singh, Niharika Chahal, Kamal Kumar

**Affiliations:** ^1^Department of Veterinary Public Health and Epidemiology, Lala Lajpat Rai University of Veterinary and Animal Sciences, Hisar, Haryana, India; ^2^Department of Animal Biotechnology, Lala Lajpat Rai University of Veterinary and Animal Sciences, Hisar, Haryana, India; ^3^Department of Veterinary Surgery and Radiology, Lala Lajpat Rai University of Veterinary and Animal Sciences, Hisar, Haryana, India; ^4^Department of Animal Genetics and Breeding, Lala Lajpat Rai University of Veterinary and Animal Sciences, Hisar, Haryana, India; ^5^Department of Veterinary Physiology and Biochemistry, Lala Lajpat Rai University of Veterinary and Animal Sciences, Hisar, India; ^6^Department of Veterinary and Animal Husbandry Extension Education, Lala Lajpat Rai University of Veterinary and Animal Sciences, Hisar, Haryana, India

**Keywords:** academic stressors, correlation, India, stress, veterinary students

## Abstract

**Background:**

Veterinary education, is a rigorous professional training program, which exposes students to significant academic and non-academic pressures. The identification of stressors and stress levels among veterinary students mighty help the designing and implementation of coping strategies to protect the students' mental health.

**Methods:**

A 44-item based cross-sectional questionnaire survey was prepared and disseminated among veterinary students in India to identify the stressors responsible, measure the amount of stress, and relate stress to characteristics like gender, degree year, and family income. A total of *n* = 611 veterinary students across 14 states including 27 colleges/universities participated in the study. The collected data was evaluated for sampling adequacy, construct validity, and reliability using a set of statistical tests.

**Results:**

The analysis revealed high sampling adequacy with a KMO value of 0.957 and a highly significant anti-image correlation (*p* < 0.001). The principal component analysis generated six factors or subscales which effectively explained 51.98% of the variance in the data, depicting high construct validity. The Cronbach's alpha value of 0.957 revealed high internal consistency for the questionnaire. Analysis revealed more than 94% of pupils under stress, with levels ranging from moderate to severe. Academic-related stressor (95.58%) was the leading cause of overall stress in the present study followed by inter- and intrapersonal and career related-stressors (93.12%) and exams and evaluation-related stressor (90.99%). In comparison to male students, female students reported significantly higher levels of overall stress, academic stress, and intrapersonal and interpersonal stress (*p* < 0.001) using Chi-square. The students from lower-income families experienced significantly higher overall stress as well as stress due to family responsibilities (*p* < 0.001). The first-year undergraduate students reported significantly higher (*p* < 0.001) stress due to family responsibilities-related stressors whereas second-year students due to social activities-related stressors. The hierarchal regression model predicted that gender, family income, academic-related stressors, inter- and intrapersonal and career-related stressors, and social activities-related stressors can be employed to evaluate overall stress among students, as they ensured the maximum variance in the data (*p* < 0.001).

**Conclusions:**

To the best of our knowledge, this is the first Indian study to identify stressors, quantify associated stress and predict major attributes to be targeted in future studies for veterinary students.

## Introduction

Stress is a non-specific response of the body to the various events which enable individuals to perceive and cope with the adversities in both physical and social environments (1). The events leading to stress including personal and environment are considered stressors. The response of an individual to a stressful stimulus may be complex and is generally attributed to various factors *viz*., socio-cultural, psychological, physical, environmental as well as individual perception (2).

Veterinary education is a rigorous professional degree program with a heavy workload that demands significant personal time (3). Although challenges are part of every professional curriculum and the subsequent phases of life transition, veterinary education has its pattern of situations that may cause students to feel tense and overwhelmed (4). For instance, the persistent comparison with medical counterparts within initial days of admission, maintenance of good academic scores for fellowship, lower stipend for internships and conflicts with animal owners, etc. are more frequent events for veterinary students in India. This challenging environment poses a serious concern for the mental well-being of veterinary students and needs to be addressed in terms of impact, prevalence, and management (5). Academic stressors are majorly responsible for adverse outcomes such as mental outbursts, stress, depression, and anxiety among veterinary as well as medical students (6). The heavy workload, lack of clinical skills, concerns of being compared with peers, lack of time for studies, and recreational activities and ignorant administration are some of the frequently reported sub-factors under academic stressors leading to stress among veterinary students (4, 7). A comparative study by Yang et al. (8) in Australia reported higher overall stress among veterinary students as compared to medical students.

Other than academic stressors veterinary students are also equally prone to non-academic stressors, including family issues or relationships, personal health concerns, and financial problems (7). These stressors may pave the way to mental health problems such as debilitating stress, anxiety, and depression among students, in absence of coping strategies (6, 9). The inter- and intrapersonal and professional interactions as well as conflicts between animal and human interests also predispose veterinary students to mental stress (2, 10). These conflicts may arise due to the owners' perception of lack of effect after treatment, non-cooperation of owner in the restraining of their pet/animal, and pressure on students for treatment using influential position (11). The lack of career opportunities along with insulting remarks by politicians in recent times in India may add to the list of stressors veterinary students are subjected to (12). Additionally, students in the pre-clinical and clinical year of graduation and postgraduate (PG) students involved in clinical practice at university/college clinics are more likely to experience emotional aspects while dealing with various aspects of the death of the animals, managing challenging clients (angry or non-cooperative), concerns over lack of time for recreation, and mistakes in diagnosis (2). These practice-related events may impart a long-term mental impact on veterinary students leading to other unfortunate consequences like suicidal tendencies (13).

The stress experienced at the graduation level may expand into the professional lives of veterinarians as practitioners and academicians leading to mental health consequences that might affect the quality of life and reduces the socioeconomic productivity (5). The norms, beliefs and behavior of a professional expressed in life are influenced by student life experiences during their professional education (14). There is thus a need to provide such an atmosphere that teach and suggests practical approaches for enhanced resilience in response to stress.

A manageable level of stress for a sensible duration may however serve as an important stimulant to meet life's challenges but chronic stress can have serious consequences on the emotional, psychological, and physical health of an individual (15). Several studies concerning various stressors and associated stress among veterinary students have surfaced in recent times worldwide, but unfortunately, similar efforts are still lacking in Asian countries including India. Although a few studies describing the mental health issues among medical students in India (16) have been conducted but the veterinary counterparts were completely ignored as there is not a single study investigating the issue among veterinary students.

The detection of stress and associated stressors at early stage might help in reducing the risk of psychosocial and behavioral problems, such as violence, suicidal thoughts, and use of drugs and alcohol as coping method (17, 18). It will also help in developing coping strategies or approaches to reduce or eliminate the most daunting stressors and thus assurance of quality life for the individual. Therefore, this study aimed to develop and validate a new survey instrument to identify the stress and associated stressors among veterinary students in India and to determine relationship of stress with other variables *viz*., gender, income, and degree year. The study further aimed to provide the veterinary students with a validated self-reporting tool to assess the level of stress they are subjected to and to seek help accordingly. Findings of current study are expected to provide a base study to attract the attention of health planners and veterinary institutes administrations to develop sufficient stress coping programs. The findings will provide most important and frequent stressors to be targeted to reduce the overall stress to the veterinary students in India. Based on literature search and to the best of our knowledge, this is the first Indian study of its kind.

## Materials and methods

### Study design

The students enrolled in various veterinary universities/colleges of India in undergraduate (UG); Bachelor of Veterinary Sciences and Animal Husbandry (BVSc & AH), post-graduate (PG); Master of Veterinary Sciences (MVSc), and Doctor of Philosophy (Ph.D.) degree courses at the time of the study were included in the survey. The students who have already completed 6 months or more in the veterinary curriculum were included and the students already graduated were excluded from the survey to avoid confounding bias. Participation in the study was voluntary, and written consent was obtained from all the participants in the study. The sample size was calculated using the “Raosoft calculator” (http://www.raosoft.com/samplesize.html?nosurvey). The sample size of 377 was estimated based on a 50% response distribution, a 5% margin of error, and a 95% confidence interval. The expected response proportion of 50% was assumed as there was no such study earlier.

### Sampling and procedure

A total of 1,000 questionnaires were to be sent to students of different veterinary institutes in India through emails and/or personal contacts and social media groups. For this purpose, an academician (teacher/researcher) or post graduate students (known to researchers) from each veterinary institute of India was contacted and was requested to share the questionnaire pdf file with students of their respective institute (snowball sampling). The questionnaire pdf file included a consent form and a link to the online interface of Google Forms (Google LLC, Mountain View, CA, USA). The survey remained open for 3 months from July 2021–September 2021.

### Survey instrument

For the present study, a cross-sectional questionnaire was designed with 50 items initially. After formal testing on 20 undergraduate (UG) students, six items were removed due to irrelevant and controversial nature (admission quota, caste, worst incidents of academic life and abuse by teachers, etc.), leading to a final set of 44 closed items (Supplementary Table 1). The items were organized into six categories *viz*., academic-related stressors (ARS), inter or intrapersonal, and career-related stressors (IPCRS), teaching and learning-related stressors (TLRS), exams and evaluation-related stressors (EERS), social activities related-stressors (SARS) and family responsibilities related stressors (FRS), based on the extensive review of related veterinary and medical literature (16, 19, 20). The first section of the questionnaire collected socio-demographic data of students specifically gender, age, religion, family income, degree year, state of residence, and name of the currently enrolled college/university. The second section included questions based on rating the intensity of stress caused by each item on a Likert scale of 0 (causing no stress) to 4 (causing extreme stress) (21). For example, “How much stress heavy workload causes to you?” with a response option on the scale (0-no stress at all; 1-causes mild stress; 2-causes moderate stress; 3- causes high stress; 4- causes severe stress). The items were placed in random order to avoid the carryover effect of responses. The survey instrument was designed to take 10–15 min to complete. The scoring of values for quantification of stress level was done by obtaining the mean values for each of the categories/subscales and the scores were rated as no or mild stress (0.00–1.00), moderate (1.01–2.00), high (2.01–3.00), and severe (3.01–4.00) as described by Jayarajah et al. (20). The overall stress was calculated by taking the mean of the overall score of each category.

### Data analysis

The completed questionnaires were manually checked for data quality and codes were assigned to responses in Microsoft^®^ Office Excel 2010 for statistical analysis. The statistical analysis was performed using the Statistical Package for Social Science (SPSS) v. 20. The questionnaire items were analyzed for validity, reliability, association between the variables, and regression modeling. The questionnaire utilized exploratory factor analysis to determine whether new factors (subscales) could be created and to examine the structure of the relationship between questionnaire items. The principal component analysis (PCA) with Promax rotation and scree plot was used to extract the factors with an Eigenvalue of more than one. The correlation analysis was used to examine the independence of the new factors as a measure of construct validity. The internal consistency and reliability of the questionnaire, factors, and items were measured using Cronbach's alpha (22). The level of stress along various domains such as gender, income, professional degree year, and age was analyzed using the Chi-square test. The correlation matrix was run to study the inter-item correlations of all factors as well as non-demographic variables. The anti-image correlation, the Kaiser–Meyer–Olkin (KMO) test, and Bartlett's test of sphericity were used to determine the sampling adequacy and to assess the factorability of the correlation matrix. To justify factor analysis Bartlett's test should be significant and KMO values should exceed 0.60 (23). A series of hierarchical regression analysis were conducted to investigate which stressors significantly predicted the overall stress among the students. For hierarchical analysis, the categorical variables were provided with dummy codes (for example, dummy coding for male = 1 and female = 2). The variables that either yielded significant association or were hypothesized to be associated with stress were used in regression analysis. The gender was entered in step 1, degree year in step 2, family income in step 3, and the six stressors (ARS, IPCRS, TLRS, EERS, SARS, and FRS) were added in step 4. Only a single dependent variable (i.e., overall stress) was used.

### Ethical considerations

Ethical review and approval was not required for the study on human participants in accordance with the local legislation and institutional requirements. The participants provided their well-informed written consent to participate in the study.

## Results

A total of 611 veterinary students of India from 14 states covering 27 colleges/universities provided their consent to participate in the study, with a response rate of 61.1%. Table 1 concludes the participants' demographic breakdown. The mean age of respondents was 23.2 ± 2.8 years old with the majority identified as male (63.6%) respondents. A total of 247 (40.4%) students had family income below two lakhs. The majority of the participating students (67.10%) were from undergraduate (BVSc and AH) degree years, followed by MVSc students (27.33%) and Ph.D. students (5.57%).

**Table 1:** Socio-demographic characteristics of the participants (*n* = 611).

**Characteristics**	***M* (SD)**	***n* (%)**
Age (in years)	23.2 (2.8)	
Gender		
Male		388 (63.50)
Female		223 (36.50)
Religion		
Hindu		558 (91.32)
Muslim		19 (3.10)
Christian		13 (2.12)
Buddhist		10 (1.63)
Sikh		7 (1.14)
Others		4 (0.65)
Veterinary degree year		
BVSc and AH 1st year		59 (9.65)
BVSc and AH 2nd year		80 (13.09)
BVSc and AH 3rd year		98 (16.04)
BVSc and AH 4th year		78 (12.76)
BVSc and AH 5th year		95 (15.55)
MVSc		167 (27.33)
Ph.D.		34 (5.56)
Family income		
Below 2 lakhs		247 (40.42)
2–5 lakhs		191 (31.27)
More than 5 lakhs		173 (28.31)

M, Mean; SD, Standard Deviation.

### Sampling adequacy and construct validity

**Figure 1:**
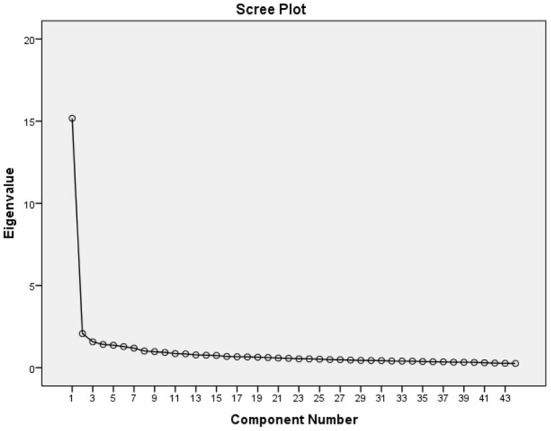
Scree plot showing Eigenvalue of the factors generated.

The KMO value for the current questionnaire was 0.957 which indicated very high sampling adequacy. Bartlett's test of sphericity was also found highly significant (chi-square = 1,222, *p* < 0.001) which again indicated that factor analysis might be useful with the current study data. The exploratory factor analysis was used to determine the construct validity of the questionnaire. The total numbers of components were extracted using PCA and Promax rotation (oblique rotation). The initial exploratory factor analysis resulted in the extraction of eight factors with Eigenvalue >1 (Figure 1) that collectively explained 56.98% of the overall variance of the data. As two out of the eight factors comprised only two items and there was shared variance between factors, items were forced to a six-factor reduction, with suppression of coefficients below 0.30. The six-factor reduction explained 51.98% of the total variance. The loading of each item to the new factors was based on the factor loading and our construct as in the method section. The factor structure along with communalities and internal consistency of each item along with factors were detailed in Table 2. The items were reshuffled to each category as loaded by the factor analysis. In this study, the scores of the items within each subscale were summed, and the mean score of each subscale was used to represent the stress level of the students in six already designed categories: *viz*., ARS, IPCRS, TLRS, EERS, SARS, and FRS.

**Table 2:** Loading of variables on factor from the Promax rotation matrix.

**Item label under factors**		**Factor** **loadings**	Communalities^*^	**Cronbach's alpha** **if an item** **deleted**
*ARS*	Rude behavior of college staff	0.785	0.572	0.882
	Ignorance of problems by the administration	0.760	0.567	0.882
	Lack of teaching skills of teachers	0.750	0.553	0.882
	Biasness/favors of teachers for selected students	0.644	0.451	0.885
	Lack of guidance regarding future jobs	0.607	0.589	0.883
	Perception of a gap in learning and practice	0.520	0.565	0.880
	Conflicts with animal owners and activists	0.487	0.458	0.886
	Lack of professional skills	0.449	0.565	0.883
	Perception of leniency in evaluations in other colleges	0.436	0.459	0.889
	Deviation of actual protocols in treatment by teachers	0.423	0.451	0.886
	Lack of guidance from teachers in the study	0.357	0.518	0.884
	Not enough study material	0.308	0.503	0.883
	Conflicts with other students	0.356	0.381	0.891
	Verbal/physical abuse by other students	0.318	0.516	0.889
*IPCRS*	Perception of failure to establish a career	0.767	0.614	0.848
	Unable to answer questions from pet owners	0.635	0.558	0.853
	Fear of getting poor marks	0.625	0.655	0.846
	Facing the death of an animal at clinics	0.599	0.408	0.865
	Lack of time for revision of content	0.573	0.607	0.851
	Need to do well (by others)	0.481	0.527	0.852
	Self-expectation to do well	0.474	0.464	0.860
	Preparing competitive exams	0.445	0.410	0.861
	Difficulty in understanding content/course	0.337	0.531	0.852
*TLRS*	Lack of recognition/praise for your work	0.657	0.536	0.828
	Frequent interruption of work by others	0.619	0.500	0.827
	Stress while working with computers	0.609	0.549	0.838
	Difficulty in finding a person to talk to	0.568	0.550	0.825
	No feedback by teachers	0.564	0.547	0.821
	Conflict with teachers	0.551	0.511	0.821
	Unable to answer in class	0.431	0.599	0.822
	Lack of communication among teachers	0.412	0.570	0.824
	Self-Unwillingness to study veterinary science	0.318	0.342	0.839
*EERS*	The stress of examination or tests/viva	0.848	0.615	0.782
	Frequent examinations	0.697	0.591	0.779
	Heavy workload	0.663	0.567	0.770
	The stress of class presentation/discussion	0.620	0.545	0.802
	Falling behind in study schedule	0.450	0.557	0.779
	Inappropriate assignments by teachers	0.402	0.530	0.791
*SARS*	Talking to animal owner's history taking	0.722	0.584	0.578
	Fear of clinical practice (injection/surgery)	0.473	0.491	0.604
	Parental wish to study another field/profession	0.399	0.376	0.638
	Lack of time for family and friends	0.354	0.403	0.595
*FRS*	Financial problems (family debt)	0.637	0.536	0.592
	Family responsibilities/expectations	0.585	0.526	0.593

* Communalities are estimates of variance accounted for by each variable in the factor solution.

### Reliability analysis

The questionnaire showed high internal consistency indicated by Cronbach's alpha value of 0.957 which lies in an excellent grade. Moreover, Cronbach's alpha values of individual subscales ranged from 0.647 to 0.892, indicating good internal consistency (Table 3). When individual items were removed, Cronbach's, alpha ranged from 0.578 to 0.889 indicating that all the items contributed to the adequacy of the scale (Table 2). Additionally, an anti-image correlation with individual items also confirmed the sampling adequacy (*p* < 0.001) for further analysis.

**Table 3:** Questionnaire factors with mean score and reliability estimates (Cronbach's alpha).

**Factor**	**Number** **of items**	**Mean (SD)**	**Cronbach's alpha**
**ARS**	14	2.88 (0.79)	0.892
**IPCRS**	9	2.82 (0.86)	0.868
**TLRS**	9	2.46 (0.89)	0.843
**EERS**	6	2.64 (0.86)	0.813
**SARS**	4	1.95 (0.84)	0.670
**FRS**	2	2.54 (1.05)	0.647

### Correlation analysis

Inter-factor correlations, as well as correlations of factors with other variables of the questionnaire (gender, income, and degree year), were examined using bivariate variation (Spearman's rho) as shown in Table 4. Inter-factor correlations ranged between 0.006 and 0.608, which indicates generally acceptable independence. The highest correlation between ARS and IPCRS (0.608) indicates some overlap between these two factors. All the factors, except IPCRS, had a higher discriminant ability and were statistically significant (*p* < 0.05). All the factors were significantly correlated with overall stress except for FRS, and TLRS, and these two seem to have a lower discriminant ability to measure overall stress than other factors. The correlation between factors and other variables varied from 0.006 to 0.280. ARS and overall stress were significantly correlated to gender and income but not to degree year. The inter-item total correlation value was more than 0.3 for 20 items. The rest of the items, however, had a lower but statistically significant correlation and there was no considerable change in Cronbach's alpha with the deletion of any item, which indicates acceptable reliability. Therefore, all 44 items were included in the questionnaire. The Cronbach's alpha value of each stressor group was depicted in Table 3 and Cronbach's alpha value if the scale item was deleted for an individual item in Table 2.

**Table 4:** Correlation analysis of main variables (*N* = 611).

		**1**	**2**	**3**	**4**	**5**	**6**	**7**	**8**	**9**	**10**
1.	ARS	–	0.608^**^	−0.006	−0.093^*^	−0.011	0.018	0.701^**^	0.220^**^	−0.081^*^	0.063
2.	IPCRS		–	0.018	−0.010	−0.070	0.054	0.748^**^	0.280^**^	−0.008	−0.049
3.	TLRS			–	0.531^**^	0.500^**^	0.532^**^	−0.022	0.057	−0.047	−0.079^*^
4.	EERS				–	0.488^**^	0.368^**^	−0.083^*^	−0.010	−0.035	−0.069
5.	SARS					–	0.358^**^	−0.098^*^	−0.043	−0.130^**^	−0.068
6.	FRS						–	0.034	0.016	−0.114^**^	−0.098^*^
7.	Overall Stress							–	0.172^**^	−0.141^**^	−0.006
8.	Gender								–	0.197^**^	0.204^**^
9.	Income									–	0.155^**^
10.	Year										–

* p < 0.05.

** p < 0.01.

### Characterization of stress and its relation with other variables

All the students reported some degree of overall stress. The students with a mean score of more than 1.0 i.e., those who reported scores in the moderate, high, and severe categories were considered under stress. Overall, for 611 students, 94.10% (575) were under stress (mean score of more than 1.0), of which 40.3% (232) reported moderate level of stress, 47.5% (273) reported high stress and 12.2% (70) students reported the level of stress as severe. Overall among the stressed 59.65% (343/575) students had high to severe stress. The most important stressors group among veterinary students was ARS (95.58%) followed by IPCRS (93.12%), EERS (90.99%), TLRS (87.72%), FRS (79.54%) and SARS (66.12%) as shown in Table 5. In summary, Table 6 showed that female students significantly experienced more overall stress with ARS and IPCRS being the major contributing stressors as compared to the male students. The students with lower family income (less than two lakhs per annum) experienced significantly higher overall stress as compared to students with higher family income. The students of the BVSc and AH second year reported SARS as a significant stressor whereas first-year students responded FRS as a major stressor.

**Table 5:** Proportion of students who indicated stress related to specific factor^*^.

**Factors**	***n* (%)**
**ARS**	584 (95.58)
**IPCRS**	569 (93.12)
**EERS**	556 (90.99)
**TLRS**	536 (87.72)
**FRS**	486 (79.54)
**SARS**	404 (66.12)

* N = 61, Mean score more than 1.0.

**Table 6:** Significant relation of gender, family income, and degree year with overall stress and individual factors.

**Variables**		**Frequency,** ***n* (%)**	***p*-value** **(Chi-square)**
**Gender**			
Overall stress	Male (389)	356 (91.51)	< 0.001
	Female (222)	219 (98.64)	
ARS	Male (389)	363 (93.31)	< 0.001
	Female (222)	221 (99.54)	
IPCRS	Male (389)	350 (89.97)	< 0.001
	Female (222)	219 (98.68)	
**Income**			
Overall stress	Below 2 lakhs (247)	234 (94.73)	< 0.001
	2–5 lakhs (191)	179 (93.71)	
	More than 5 lakhs (173)	162 (93.64)	
FRS	Below 2 lakhs (247)	208 (84.21)	< 0.001
	2–5 lakhs (191)	142 (74.34)	
	More than 5 lakhs (173)	136 (78.61)	
**Degree year**			
SARS	First-year (59)	31 (52.54)	< 0.005
	Second-year (80)	65 (81.25)	
	Third-year (98)	69 (70.40)	
	Fourth-year (78)	52 (66.66)	
	Final Year (95)	69 (68.42)	
	MVSc (167)	95 (56.88)	
	Ph.D. (34)	23 (67.64)	
*FRS*	First-year (59)	59 (100)	< 0.001
	Second-year (80)	70 (87.50)	
	Third-year (98)	88 (89.79)	
	Fourth-year (78)	54 (69.23)	
	Final Year (95)	73 (76.84)	
	MVSc (167)	121 (72.45)	
	Ph.D. (34)	21 (61.76)	

### Hierarchical regression analyses

The results of the hierarchical regression analysis indicated that in step 1, gender positively predicted and accounted for 3% of the variance (*p* < 0.001). In step 2, the degree year was not significant. In step 3, the family income negatively predicted and accounted for 6% of the variance (*p* < 0.001). In step 4, the six stressors significantly accounted for an additional 68% of the variance (*p* < 0.001), with only ARS, IPCRS significantly predicting the overall stress (Table 7).

**Table 7:** Hierarchical regression of independent variables on overall stress among veterinary students.

**Variable**	***B***	**SE *B***	**Beta**	**Sig**.	***R* ^2^**	**Adjusted *R*^2^**	**Δ *R*^2^**	***F* change in *R*^2^**
**Step 1**								
Gender	0.28	0.06	0.17^¶^	0.000	0.03	0.03	0.03	20.00^¶^
**Step 2**								
Degree Year	−0.01	0.01	−0.02	0.470	0.03	0.02	0.00	0.52
**Step 3**								
Income	−0.17	0.03	−0.18^¶^	0.000	0.06	0.06	0.03	20.78^¶^
**Step 4**								
ARS	0.36	0.02	0.37^¶^	0.000	0.68	0.68	0.62	201.05^¶^
IPCRS	0.46	0.02	0.52^¶^	0.000				
TLRS	0.02	0.02	0.02	0.405				
EERS	−0.01	0.02	−0.01	0.526				
SARS	−0.06	0.02	−0.07^ɤ^	0.008				
FRS	0.00	0.02	0.01	0.667				

* N = 611.

¶ p < 0.001.

ɤ p < 0.01.

To summarize, female students had a higher level of overall stress than male students. The students with lower family income have reported high overall stress and the students who reported high levels of ARS, IPCRS, and SARS, were more likely to be included in the high overall stress category.

## Discussion

The current study yielded six factors using principal factor analysis. All the six factors generated showed good internal consistency and explained the adequate cumulative variance in the data. Overall, of the 611 students, 575 (94.10%) were under stress. The more number of students under high to severe stress category in the current study is a serious concern. The moderate level of stress responded by students, might be necessary and manageable to provide motivation and increased productivity (24). Evidences suggest that a moderate level of stress strengthens the connection between neurons in the brain, leading to the improvement in attention span, and memory, and thus enhanced productivity (15, 25). On the other hand, high and severe stress can be detrimental to the physical and mental health of individuals leading to adverse consequences such as depression, anxiety, and suicidal thoughts (26). The current study reported ARS as a major contributor to overall stress with a maximum of 95.58% of students citing academic problems as major stressors. Williams et al. (2), observed that more than 85% of veterinary students experienced academic-related stress, including academic and practical workload, which is in line with several other studies in various countries (10, 27, 28) including the current study. Another study by Gupta et al. (16) among medical students in Kolkata, India reported that 91.1% of medical students experienced stress, and academic-related stressors were identified as a major source of stress for 94.9% of students, which is in consensuses with our findings. The academic stressors mainly included heavy workload, rude behavior of college staff, ignorance, and biases by college/university administration. Although heavy workload may also be regulated and a course work with reduced burden can be designed or implemented but the factors such as rude behavior of non-teaching staff and ignorance of genuine student problems by the administration are more serious issues as they fulminate within the wall of the institutes and easier to resolve. In the present study, female veterinary students experienced significantly higher overall stress, and ARS and IPCRS related stress as compared to male students. The similar patterns of stress among female students as compared to male counterparts are reported in various studies around the globe (6, 29–31). A set of conditions such as depression, chronic pain, stress, and anxiety disorders are more frequent among females (32, 33) and this may be partly attributed to the effects of sex hormones as some of these gender differences emerge during reproductive years and generally diminish with age or after menopause (34). Additionally, literature describes ruminative thinking as more frequent in females which increase the risk of stress and depression (35). The academic performance of female students in developing countries like India plays a decisive role in higher education and career, which might create pressure additional to regular convincing for getting married by parents which might affect their academics and other activities, and this can be the reason for high academic, inter or intrapersonal and conflict-related stress and overall stress (36). Although every stressors needs attention to safeguard the mental health of female students but it further needs investigations to know how the academic stressors are affecting the female students and the strategies to check the same.

The students with lower family income (male and female) had reported significantly higher overall stress as well as stress due to FRS, which might be attributed to additional family responsibilities and fear of failure to fulfill future expectations. Moreover, factors such as limited access to resources to alleviate stress, experiencing frequent financial distress, and unmet material needs and necessities such as food and clothing may predispose such students to stress (37). The lower-middle-class family students also reported higher stress due to teaching and learning-related stressors, social activities-related stressors and exams, and evaluation-related stressors when compared with the students of the upper class or high-income families. The uncertainty of future career opportunities and lack of funds to continue the education might be the factors leading to stress in such students. Huge amount of tuition fee, low-skill generation and failure to provide with job opportunities by various veterinary universities/institutes in India charge further deepens the level of stress faced by the financially weaker section of students.

The IPCRS-related stress was second most reported in the current study followed by ARS. The factors such as perception of failure to establish a career, fear of getting poor marks, and fear of facing the death of an animal were the major contributors to the IPCRS-related stress among veterinary students in India. Similar findings were reported by Williams et al. (2); where self-expectations and emotional distress while facing animal death were the major contributors to the stress among veterinary students. The perception of failure to establish a career after graduation needs attention of the institutes as graduating students needs to be guided of the opportunities and skill set to be generated during their academics. For the TLRS-related stress, lack of recognition for your work, frequent interruption by others, and computer working-related stress were major contributors. The study conducted by Singh et al. (38) among veterinary students of Punjab, India however contradicts the findings of computer related-stress, where very low respondents reported computer-related distress or anxiety. But the lack of recognition for work and frequent interruptions at the college/university might certainly lead to stress (39). Among the EERS-related stress, frequent examination, heavy workload, and viva or practical evaluation were major factors leading to stress. However, some anxiety or stress before viva voce examination is a natural but heavy workload and frequent examination might be reduced to ease such stress. Nahm and Chun (25) also reported frequent examinations as one of the important stressors to be addressed for the veterinary students. The theory and practical examination can be made more flexible in terms of the number of questions and type of questions asked, mainly by reducing the lengthy questions and including more of objective questions. For SARS-related stress the factors *viz*., history taking for a case presented in clinical complex, and fear of clinical practice were major contributors to stress and these factors were more significant for students in the pre-final year of the professional degree. These factors however can be mastered easily with regular practice and guidance from the professors and colleagues and of lesser concerns from the stress point of view.

On the construct and validity point, the three stressor categories of the questionnaire *viz*., ARS, IPCRS, and SARS positively predicted the overall stress (*p* < 0.01), where ARS and IPCRS were highly correlated with overall stress using correlation analysis. Other variables such as gender and family income also predicted the variance within overall stress. The hierarchical regression analysis revealed that academics, inter or intrapersonal and career-related, and social activities-related stress positively predicted the overall stress for veterinary students in India. These findings can help the authorities to emphasize these factors to design coping strategies for stress management among veterinary students in India. Additionally, future studies can target only these limited categories to shorten the tool and time for quantification of the stress among students. The construct designed and validated in this study can be used effectively with high consistency to measure the stress level among veterinary students in veterinary colleges or universities across India. Veterinary students can self-evaluate their level of stress using this instrument, which might help them to seek early interventions.

It is evident from the findings of current and other studies that a high percentage of veterinary students are feeling stressed to a level that might impact their quality of life. One of the simplest solution to stress among veterinary students is stress-management training or instructing anxiety-reducing strategies to students every year or on regular basis. These may be implemented utilizing counselors, psychiatrists, or even outreach programs within the student body (40, 41). Evidence suggests that utilization of mindfulness based programs and encouraging a growth mindset, in place of a fixed mindset might help veterinary students to handle stressful events (42). Although the strategies above seem promising, literature also provides evidence that veterinary students might not utilize the counseling and medical attention for mental health issues, subjected to a negative stigma associated with such issues (43). Further, researchers have recommended various socio-behavioral models for promoting behavior changes among students, of which the most recently proposed theoretical model; Multi-Theory Model of Health Behavior Change (MTM) by Sharma (44) incorporates multiple socio-behavioral theories, into a model of two components—initiation and sustenance of health behavior change. This model has been used already by various researcher for the prediction of initiation and sustenance of health and stress management behavior changes such as a reduction in binge-drinking, increase in eating fruits and vegetables, improvement in sleep behaviors, increased physical activity, portion size control and participation in conscious relaxation behaviors (5, 45, 46). The MTM might be employed for predicting initiation and sustenance of health behavior change as it is both specific for health education and sustainable for long-term change. Another study found that a life coach for first-year medical students helped in the improved mental health of the students (47), which might be utilized by veterinary institutes to promote an intervention targeting behavioral confidence.

The professional faculty in veterinary institutes has a responsibility to support students during their education and to prepare them for all aspects of professional life and they can help address this problem by educating with appropriate knowledge, fostering the development of necessary skills, and providing guidance and empathetic support when needed (27). The administration needs to be more accessible and empathetic toward students' problems rather than ignoring them. As much of the stress reported in the study arises from academic-related stressors including heavy workload and intensive curriculum, there is an urgent need to introduce some newer changes to design a more apt and less stressful curriculum. The inclusion of workshops and meditation programs in the veterinary curriculum for stress management might serve as a guiding tool to balance life and raise awareness of individual needs (27). The cultural activities, sports activities, and sessions as workshops seeking feedback from the students from time to time might help to create a culture of inclusion, support, and acceptance.

Based on his study, Siqueira Drake (48) suggests the promotion of therapeutic or skill development groups to provide the students a platform to discuss their issues or to gather some skills, where potential topics to discuss may be time management, stress coping strategies, managing personal and professional relationships, and communication skills. These interventions thus might help by creating a supportive environment for students, where they can feel comfortable to seek the help if needed.

The administration may contribute by changing the atmosphere within the institute by arranging events, during the first degree year and beyond, in such a way to better connect students with support staff, teachers, and one another. Researchers across the globe consider that college administrators and faculty should create a safe, non-threatening environment to use resources such as counseling for mental-health concerns (49). To the best of our knowledge, this is the first study designed and validated to map out the stress among veterinary students in India.

### Future directions and implications

Future investigations should be focused on identification of specific mental-health issues during the course of veterinary education. The other parameters such as depression and anxiety should also be evaluated to find any correlation with overall stress and other factors. As a higher number of students reported high to severe stress the future investigations should evaluate the coping strategies being used by these students and whether the strategies are adequate or leading to any maladaptive outcomes. Regarding educational interventions, subject or topic wise investigations need to be undertaken so as to identify the most difficult subjects for students and thus change in the content and timing can be implemented. Once these investigations yield outcomes they must be shared with authorities responsible or university managers to implement the coping strategies or programs. As the mental health nowadays is being assessed from a broader perspective, the results will surely demand the call for educational interventions which consequently will ensure improved health and life for the veterinary students.

### Limitation of the study

The low response rate from some of the veterinary colleges seems to limit the more accurate results, which could be obtained by involving every veterinary college/university in India. Further, the study was based on a single parameter of stress and other components such as anxiety and depression might be included in further studies. However, specific area selection bias can be there in the study, but sampling adequacy analysis and the first insight nature of the study might overcome that. Moreover, social desirability bias cannot be ignored but providing a real name was kept optional to reduce such bias.

## Conclusion

High overall stress among veterinary students of India is a serious concern and most of this stress is due to various academic stressors which fulminate within the parameter of veterinary colleges/universities' campuses and classrooms. There is thus an urgent need to address such issues and design coping strategies to minimize the stress among veterinary students. This study can be taken as a basis for further evaluation of stressors and the levels of stress among veterinary students in India. This study also showed that this construct with six factors had acceptable psychometric properties and is a valid and reliable instrument that can be used in local settings for the assessment of stress among veterinary students.

## Data availability statement

The original contributions presented in the study are included in the article/Supplementary material, further inquiries can be directed to the corresponding author.

## Ethics statement

Ethical review and approval was not required for the study on human participants in accordance with the local legislation and institutional requirements. The patients/participants provided their written informed consent to participate in this study.

## Author contributions

Conceptualization: KG, VJ, DP, and RK. Methodology: KG, DP, and VJ. Formal analysis and investigation: KG, YB, DY, LS, NC, and KK. Writing—original draft preparation: KG. Writing—review and editing: KG, VJ, DY, and KK. Supervision: RK and VJ. All authors contributed to the article and approved the submitted version.

## Conflict of interest

The authors declare that the research was conducted in the absence of any commercial or financial relationships that could be construed as a potential conflict of interest.

## Publisher's note

All claims expressed in this article are solely those of the authors and do not necessarily represent those of their affiliated organizations, or those of the publisher, the editors and the reviewers. Any product that may be evaluated in this article, or claim that may be made by its manufacturer, is not guaranteed or endorsed by the publisher.
