# Supplementary material for: Identification and assessment of stress and associated stressors among veterinary students in India using a cross-sectional questionnaire survey
Source: Front Public Health. 2022 Nov 21;10:1059610. doi: 10.3389/fpubh.2022.1059610 (PMC9720151; doi:10.3389/fpubh.2022.1059610)
Supplement: Supplementary file 1 [file Table_1.DOCX]

Questionnaire for Veterinary Students in India

PART A

Personal Information

Name and Admission Number…………………………………….

Permanent Address: Village/city/town……………………………District………………. State…………………….

Gender………………….

Professional Degree year……………………………………………………………

Religion……………………………………………………………………….

Annual Family Income …………………………………………………….

Glossary:

Verbal abuse is defined as to speak insultingly, harshly, and unjustly about a person.

Physical abuse is defined as to treat in harmful, injurious, or offensive way to a person.

Before you start answering: Stress is generally defined as the body's nonspecific response or reaction to demands made on it, or to disturbing events in the environment. In simple words, stress is a feeling of emotional or physical tension which may come from any event or thought that makes you feel frustrated, angry, or nervous. Stress which promotes and facilitates learning is called ‘favourable stress’ and stress which inhibits and suppresses learning is called ‘unfavourable stress’. The same stressors may be perceived differently by different medical students, depending on their cultural background, personal traits, experience and coping skills.

Image along with every item of the questionnaire along with the five choices to be filled from


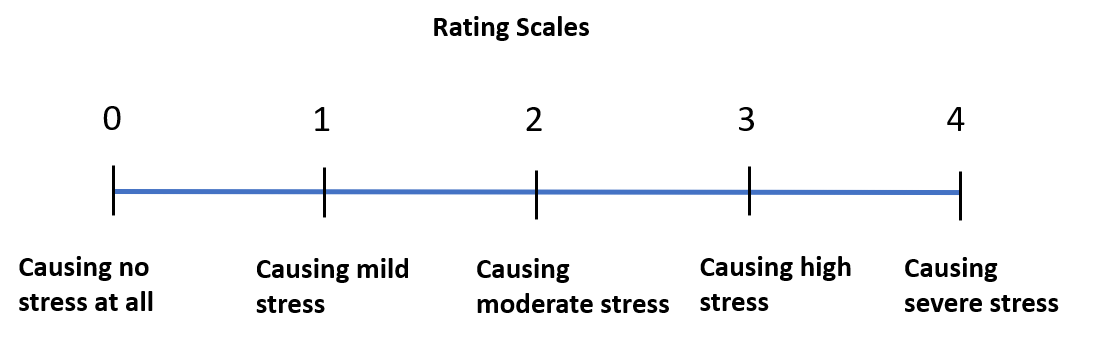


On a scale of 0 to 4, where

0= No stress at all, 1= Causing mild stress, 2= Causing moderate stress, 3= causing high stress, 4= causing severe stress

For example:


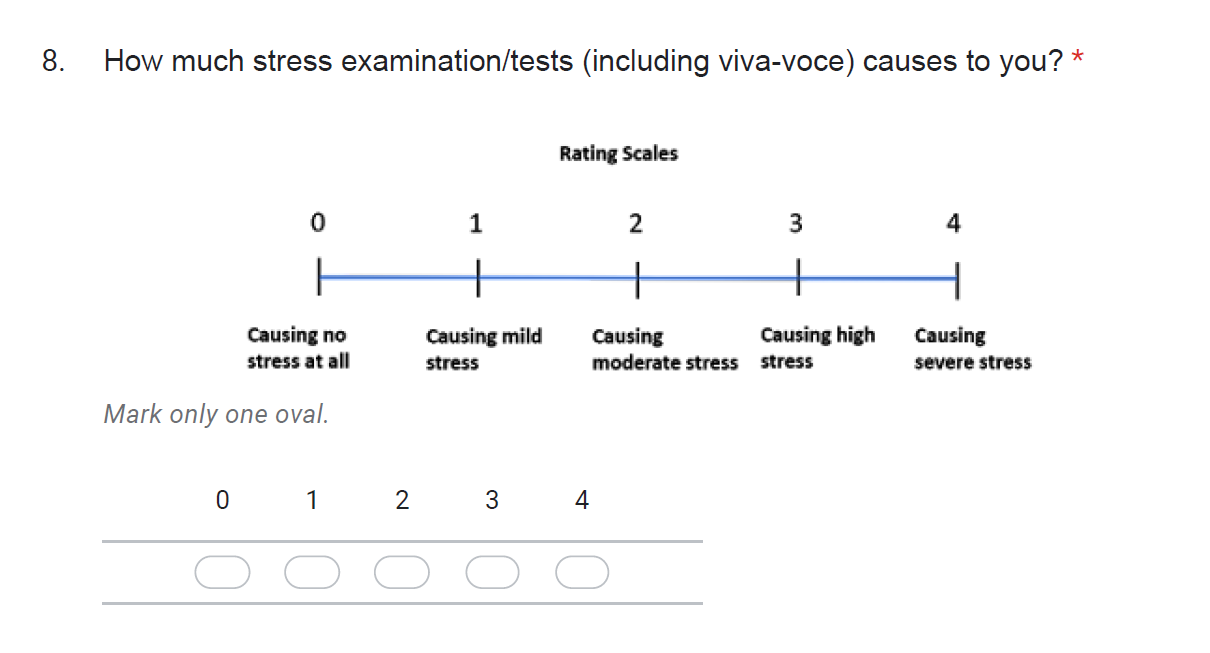


Based on the rating scale and example above answer the following questions:

PART B

| 1. How much stress examination/tests (including viva-voce) causes to you? |
| --- |
| 1. What amount of stress it causes when you have conflict (a serious disagreement or argument) with other students? |
| 1. How much you feel stressed by family responsibilities (expectations etc.)? |
| 1. If in case, clinical subject teachers deviate the treatment protocol taught to you (in Theory classes) during actual treatment of animals, how much stress you feel about this on professional/ethical ground? |
| 1. How much stress it causes to you when teachers are biased to your certain colleagues (favouring in marks/ clinical practice opportunity) ? |
| 1. How much stress it causes when there is lack of guidance regarding future/ job opportunities/ conferences/ skill development etc. by department/University? |
| 1. How much stressed do you feel due to lack of important professional skills even after graduation/post-graduation? |
| 1. How much stressed do you feel due to rude/ignorant attitude of college staff/lab attendants towards you? |
| 1. How much stressed you feel when your problems are frequently ignored by University/ college/ Hostel administrations even after bringing it to their notice? |
| 1. How much stressed you feel by the perception that there is huge gap in veterinary science you learned in college and actual being practiced in the field? |
| 1. How much stress is caused by lack of teaching skills of teacher? |
| 1. How will you rate the stress caused to you when you get into the conflict (a serious disagreement or argument) with animal owners/ animal welfare activists due to their inappropriate behaviour towards you/ veterinarians? |
| 1. If you come to know that the certain Universities/ colleges tend to be more lenient in examination evaluation as compared to your university/ college then how much stress you feel about it? |
| 1. How will you rate the stress caused to you while talking to animal owners during history taking (in clinics). |
| 1. The amount of stress caused due to verbal or physical abuse by other students? |
| 1. The stress caused by parental wish/forcing to study the field other than veterinary science? |
| 1. How much stress you feel due to self-expectation/need to do well? |
| 1. When you find that there is not enough study material available with you for study, what level of stress it causes to you? |
| 1. What amount of stress heavy workload causes to you? |
| 1. The stress caused by Participation in class discussion or during presentation (in front of class/audience). |
| 1. The stress caused by Falling behind in reading/studying schedule? |
| 1. What amount of stress is caused by Lack of guidance from teacher (s)? |
| 1. Stress caused by fear of clinical practice (surgery/injections/treatment). |
| 1. Stress caused by Lack of time for family and friends. |
| 1. Stress caused while preparing for competitive (UPSC/ICAR/ NET) entrance test examinations (? |
| 1. Rate the stress caused if you are unable to answer the questions from animal/pet owners? |
| 1. Rate the stress caused by assignments given by teachers which you consider inappropriate. |
| 1. Rate the stress caused when you are facing difficulty in understanding the content (course/subject) ? |
| 1. Stress level you feel if you face illness/death of animal in front of you? |
| 1. Rate the stress caused by fear of getting poor marks/grade in exams despite studying. |
| 1. Rate the stress caused by perception that you will fail to establish a career due to a lack of competence. |
| 1. What stress it causes to you when you have no time for revision/review well before exams. |
| 1. What level of stress it causes to you when you are having difficulty in finding a person to talk to about your problems? |
| 1. What amount of stress it causes when your work is frequently interrupted by others? |
| 1. Your teacher asked some questions to you in class, and you are unable to answer the questions from the teachers. What level of stress it causes to you? |
| 1. Rate the stress caused to you if there is conflict with teacher (s) on some topic/discussion. |
| 1. What stress do you feel due to self-Unwillingness to study veterinary science discipline? |
| 1. What stress is caused by Need to do well (if imposed by others)? |
| 1. You find that teacher is just teaching and not taking enough feedback. How much stress it causes? |
| 1. You did some excellent work, and you got no recognition/praise for your work. What level of stress is caused by this? |
| 1. What level of stress you feel while working with computers? |
| 1. How much stressed you feel due to your financial problems (family debt/economic problems)? |
| 1. What amount of stress is caused to you by lack of communication/ coordination among university/department teachers? |
| 1. Rate the stress caused by frequent examinations. |
